# Effective cochlear implantation for idiopathic hypertrophic pachymeningitis with bilateral profound hearing loss: A case report

**DOI:** 10.1097/MD.0000000000036124

**Published:** 2023-11-24

**Authors:** Katsuyoshi Idei, Mitsuru Kitamura, Marie N. Shimanuki, Makoto Hosoya, Nobuyoshi Tsuzuki, Natsuki Hasebe, Takanori Nishiyama, Hiroyuki Ozawa, Naoki Oishi

**Affiliations:** a Department of Otorhinolaryngology, Head and Neck Surgery, Keio University Hospital, Tokyo, Japan.

**Keywords:** bilateral profound hearing loss, cochlear implant, idiopathic hypertrophic pachymeningitis

## Abstract

**Rationale:**

Hypertrophic pachymeningitis (HP) is a local or diffuse fibrous thickness of the dura mater of the brain or spinal cord, caused by infection or connective tissue disease. Headache is the most common clinical symptom, followed by various cranial nerve disorders such as visual impairment, diplopia, and hearing loss. HP can be classified into secondary and idiopathic. Here, we report a case of bilateral progressive profound sensorineural hearing loss diagnosed in a patient with idiopathic HP, where a cochlear implant was effectively used.

**Patient concerns:**

The patient was a 77-year-old woman. Hearing loss gradually progressed bilaterally, and magnetic resonance imaging showed a space-occupying lesion with a continuous contrast enhancement in the bilateral internal auditory canals, and diffused dural thickening from the middle to the posterior cranial fossa.

**Diagnoses:**

A trans-labyrinthine biopsy was conducted, and a definite diagnosis of idiopathic HP was made. Thickening of the dura mater in the bilateral internal auditory canals was thought to cause profound hearing loss.

**Interventions and outcomes:**

A cochlear implant was implemented 4 months after biopsy, and a favorable hearing response was obtained postoperatively.

**Lessons:**

This is the first report of a cochlear implant in a patient with idiopathic HP. Cochlear implantation was considered a good treatment for profound hearing loss due to idiopathic HP, which provides a reference for patients to receive timely and correct treatment.

## 1. Introduction

Hypertrophic pachymeningitis (HP) is a local or diffuse fibrous thickness of the dura mater of the brain or spinal cord, caused by infection or connective tissue disease.^[1]^ This disease concept was first proposed by Charcot et al in the late 19th century^[2]^ and can be classified into secondary HP and idiopathic HP. The causes of secondary HP can be roughly divided into infectious causes, such as tuberculosis, fungi, and syphilis, and immune-mediated causes, such as an anti-neutrophil cytoplasmic antibody (ANCA)-related disease, IgG4-related disease, rheumatoid arthritis, sarcoidosis, and temporal arteritis. Although the frequency is low, tumor-based causes, such as dural metastasis of malignant tumors and malignant lymphomas, are also possible. Headache is the most common clinical symptom, followed by various cranial nerve disorders such as visual impairment, diplopia, and hearing loss. There are also reports of higher functional impairments such as disturbance of consciousness, convulsions, and memory impairment. A diagnosis of idiopathic HP is made if secondary diseases such as infections, autoimmune disease, or tumor-related causes are ruled out. Steroids are the most effective treatment for secondary HP.^[1,3]^

Here, we report a case of bilateral progressive profound sensorineural hearing loss diagnosed in a patient with idiopathic HP, where a cochlear implant was effectively used.

## 2. Case report

The patient was a 77-year-old woman. She became aware of her tinnitus 6 years ago, but pure tone audiometry revealed a normal hearing level. The following year, she noticed a left-sided hearing loss, she was diagnosed with sudden hearing loss, and was treated with oral steroids, which improved her left hearing. However, her left-sided sensorineural hearing loss worsened 5 years later, and magnetic resonance imaging (MRI) revealed bilateral internal auditory canal-space-occupying lesions. The patient was referred to our department.

**Figure 1.**
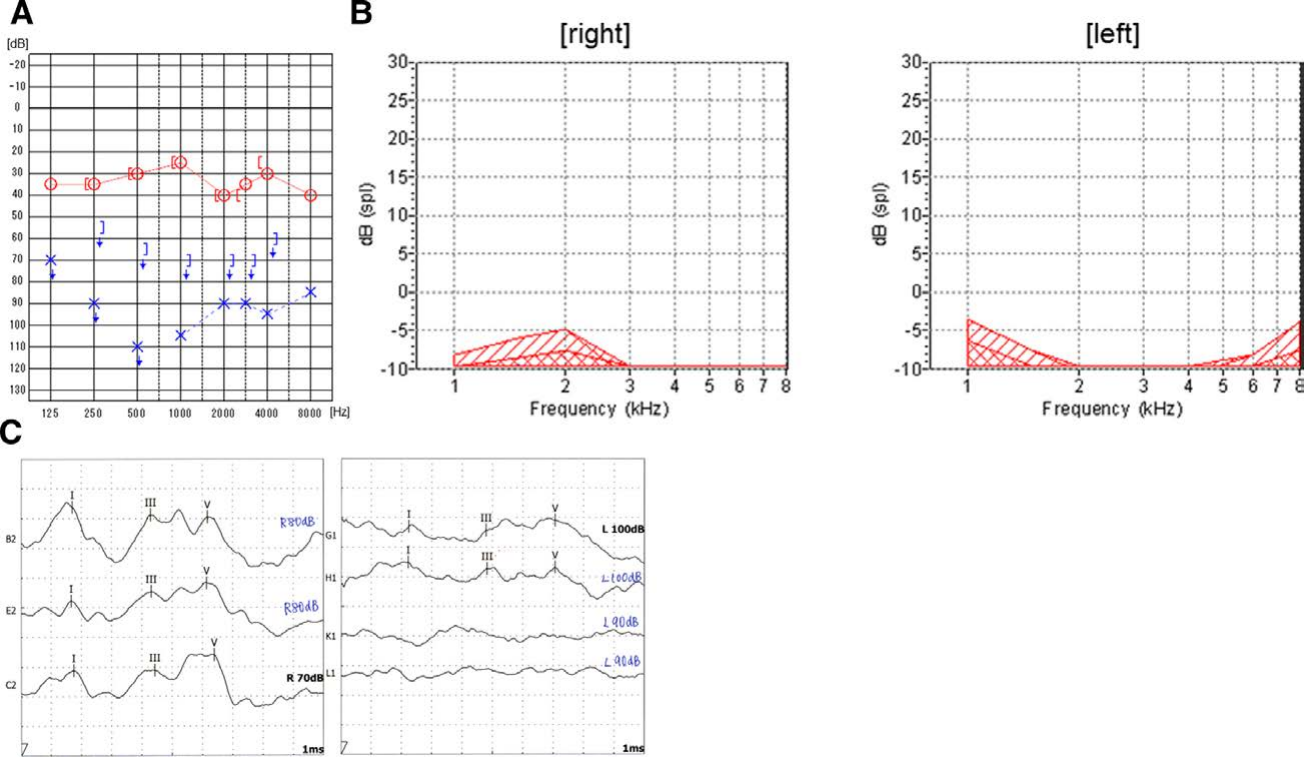
Audiological tests at the first visit to our department. (A) Pure tone audiometry showed profound hearing loss on the left. (B) The OAEs were poorly resolved bilaterally. (C) The ABR showed prolonged latencies of waves I to V on the left side. ABR = auditory brainstem response.

**Figure 2.**
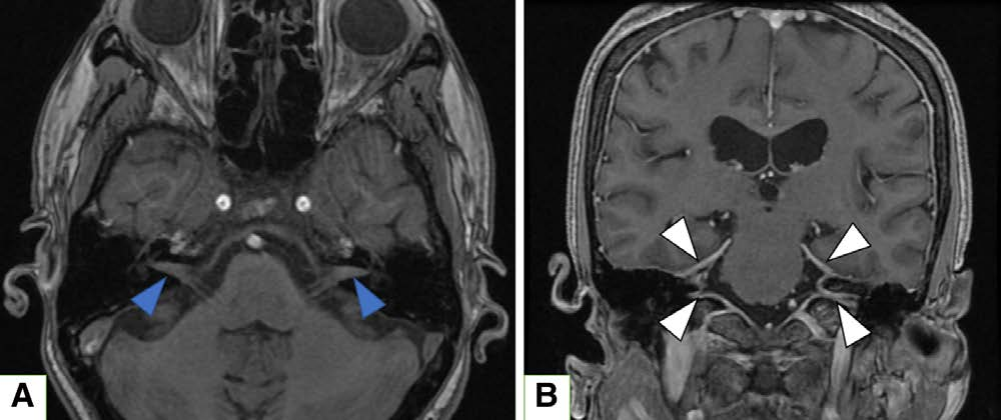
Head contrast-enhanced magnetic resonance imaging (MRI). (A) Occupying lesions in the bilateral internal auditory canals (blue arrows) (Fast Imaging Employing Steady-state Acquisition) (B) Diffuse dural thickening from the middle to the posterior cranial fossa (white arrow) (Contrast-enhanced T1-weighted Images).

The hearing level in pure tone audiometry was 30 dBHL on the right, and scaled out on the left (Fig. 1A). The best speech intelligibility in speech audiometry was 100% at 70 dB on the right, and 0% at 100 dB on the left. The otoacoustic emissions were poorly resolved bilaterally, and the auditory brainstem response showed prolonged latencies of waves I to V on the left side (Fig. 1B and C). The caloric test showed normal vestibular function bilaterally. Electroneurography of the facial nerve was 70% on the left/right, with a slight decrease on the left side. Contrast-enhanced MRI showed diffuse dural thickening from the middle to the posterior cranial fossa, and space-occupying lesions with a continuous contrast effect in the bilateral internal auditory canals (Fig. 2). Blood tests showed an elevated erythrocyte sedimentation rate and C-reactive protein level. Still, no tuberculosis, syphilis, or fungal infections were found. Additionally, immunological tests for myeloperoxidase anti-neutrophil cytoplasmic antibody and PR3-ANCA were normal, and no increase in IgG4 or angiotensin I-converting enzyme was observed. Therefore, ANCA-related vasculitis, IgG4-related disease, and sarcoidosis were not positively suspected. Low white blood cell and hemoglobin levels were observed, and aplastic anemia was diagnosed after a detailed examination at the Department of Hematology.

Given the risk of postoperative infection due to aplastic anemia, we did not conduct a biopsy of the inner ear canal and dura mater. The patient was introduced to a hearing aid for the right ear, and was followed up with audiometry and MRI periodically. Afterward, sporadic dizziness attacks began to appear, and hearing on the right side gradually worsened. Right sensorineural hearing loss progressed rapidly, and both sides were almost scaled out. To make a definite diagnosis, a biopsy was conducted using the trans-labyrinthine approach for the dural thickening and inner auditory canal-space occupying lesions on the left side.

**Figure 3.**
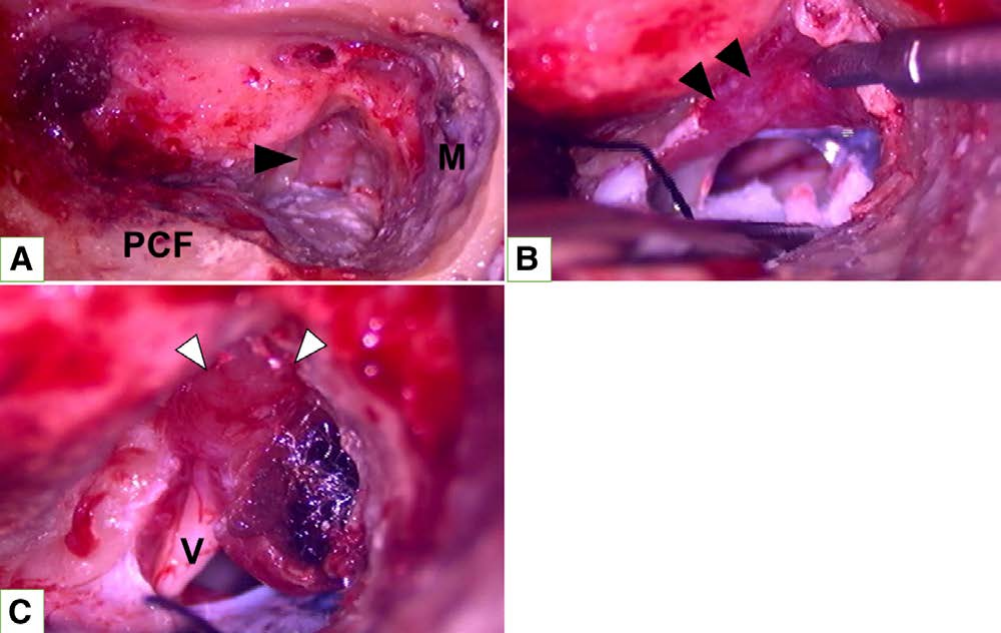
Intraoperative findings. (A) Slightly reddish dura mater of the internal auditory canal (black arrows). M: middle fossa dura, PCF: posterior cranial fossa. (B) Red jelly-like tissue just underneath the dural surface of the posterior cranial fossa (black arrow). (C) Space-occupying lesion in the inner ear canal (white arrow). V: vestibulocochlear nerve.

**Figure 4.**
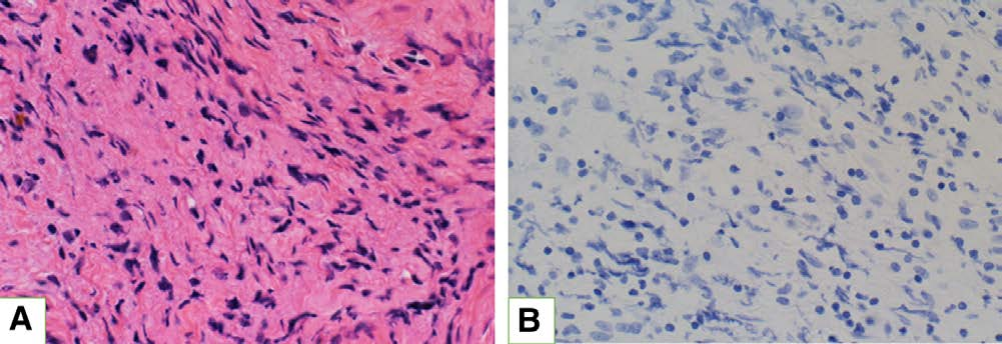
Pathological findings in the dura of the inner ear canal. (A) Hematoxylin–eosin stain × 400. There was only inflammatory cell infiltration, mainly lymphocytes, and no vasculitis or other findings of suspected secondary disease. (B) Immuno-globulin G4 stain × 400. IgG4 staining was negative.

Intraoperative findings showed a slightly reddish dura mater of the inner auditory canal (Fig. 3A), and a red jelly-like soft tissue was found just underneath the dura mater of the posterior cranial fossa (Fig. 3B). Tumor-like mass was found in the internal auditory canal along the vestibulocochlear nerve (Fig. 3C). Histopathological diagnosis of the lesions showed only inflammatory cell infiltration, mainly composed of lymphocytes, and no findings suggestive of secondary disease such as vasculitis were found (Fig. 4); therefore, the patient was diagnosed with idiopathic HP.

**Figure 5.**
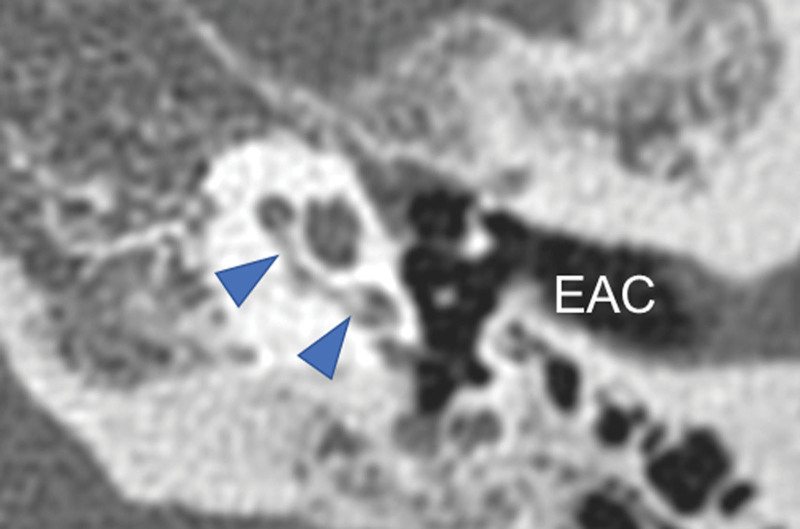
Temporal bone computed tomography, preoperative computed tomography revealed the ossification in the basal turn of the cochlea of the left ear (blue arrows). EAC: external auditory canal.

In the present case, the cause of hearing loss was thought to be due to the constriction of the cochlear nerve resulting from the dura thickening in the bilateral internal auditory canals. However, intracochlear calcification was already observed on CT before the biopsy (Fig. 5), which was thought could also cause hearing loss. A left cochlear implant was implemented 4 months after biopsy. There was intracochlear proliferation and stenosis of soft tissue at the time of operation, and a cochleostomy was needed to insert the entire electrode length (SYNCHRONY FLEX28, MED-EL). The histopathological diagnosis of the soft tissue proliferating in the cochlea was only hyalinization and calcification. As with the dura mater of the inner auditory canal, no findings were suggestive of secondary HP.

Postoperative cochlear implant threshold tests showed a favorable hearing response of 40 to 50 dB, and the hearing level has been maintained for 3 years.

Informed consent has been obtained from the party concerned for publication of case reports.

## 3. Discussion

We experienced an idiopathic HP diagnosis following a biopsy accompanying bilateral progressive profound sensorineural hearing loss. Cochlear implantation was a useful method to overcome hearing loss in idiopathic HP.

HP is a rare disease characterized by thickening of the dura mater, caused by various etiologies. Many patients are aware of chronic headaches at onset,^[4]^ and 48% have vestibulocochlear neuropathy.^[5]^ Idiopathic HP is usually not associated with elevated angiotensin I-converting enzyme, myeloperoxidase anti-neutrophil cytoplasmic antibody, PR3-ANCA, rheumatoid factor, and antinuclear antibodies. Tests for Lyme disease, syphilis, tuberculosis, and fungi must be negative. Cerebrospinal fluid examinations by lumbar punctures should be negative for infection or malignancy.^[6]^ Idiopathic HP infers that the cause of HP is unknown. Although the pathogenic mechanism is largely unknown, it is generally thought that the inflammatory infiltrate consisting of B and T lymphocytes activates fibroblasts and induces collagen deposition, resulting in tissue hypertrophy and dural thickening. It has recently been suggested that a subset of this disease is IgG4-related diseases. Symptoms depend on the inflammatory lesion’s site and the adjacent neural structures’ compression.^[7]^

In the present case, the sensorineural hearing loss was thought to be mainly caused by a retrolabyrinthine lesion, which causes auditory disturbances due to the entrapment of nerves in the inner auditory canal by the thickened dura mater. Furthermore, cochlear dysfunction was also considered a factor in hearing loss because intracochlear calcification was observed before surgery; therefore, favorable results were obtained with cochlear implantation. According to Bovo et al, HP-induced cochlear damage is due to compression of the inner ear artery and an autoimmune mechanism.^[3]^ Acoustic neuroma, a lesion of the 8th cranial nerve, is known to cause inner ear hearing loss due to tumor-induced ischemia and biochemical abnormalities in the inner ear fluid. Kawamura et al reported that a case of bilateral sensorineural hearing loss associated with HP in Cogan disease had a sense of pitch in the promontory test, and discussed that cochlear damage with a mechanism similar to that of acoustic neuroma might be the leading cause of hearing loss.^[8]^ Iwasaki et al found that HP patients with dizziness and hearing loss exhibited normal otoacoustic emissions and the presence of bilateral wave I prolongation in auditory brainstem response, therefore considering the possibility of inner ear hearing loss, and citing the obstruction of blood flow to the 8th cranial nerve due to dura mater thickening as a reason for this.^[9]^

Regarding cochlear implant surgery, inserting electrodes through the typical round window is difficult when there is intracochlear stenosis, as in the present case. Pasanisi et al and Kawamura et al used osteotomy and electrode insertion in patients with cochlear plantar rotation insufficiency with Cogan disease, and a similar approach was used in the present case.^[8,10]^

This is the first report of cochlear implantation for idiopathic HP. Cochlear implantation was an effective treatment for profound hearing loss in idiopathic HP. At present, no definitive treatment method has been shown to be effective for severe to profound hearing loss caused by idiopathic HP. Hearing aids are an option, but with severe to profound hearing loss the chances of getting a meaningful auditory response are unfortunately low. Cochlear implantation is expected to improve the quality of life of patients with hearing loss due to HP, as shown in this case.

A limitation of this study is that it only reports one case. Therefore, whether cochlear implants will be effective in all cases of hearing loss due to idiopathic HP cannot be concluded. To this end, more cases need to be accumulated to determine the efficacy of cochlear implantation for hearing loss due to idiopathic HP.

## 4. Conclusions

A patient with HP and bilateral internal auditory canal lesions presenting with bilateral progressive profound sensorineural hearing loss underwent a biopsy using the trans-labyrinthine method, and a definitive diagnosis of idiopathic HP was made. Cochlear implants can be helpful for profound hearing loss due to idiopathic HP.

## Author contributions

**Conceptualization:** Mitsuru Kitamura, Naoki Oishi.

**Data curation:** Katsuyoshi Idei.

**Investigation:** Marie N. Shimanuki, Makoto Hosoya, Nobuyoshi Tsuzuki, Natsuki Hasebe, Naoki Oishi.

**Methodology:** Naoki Oishi.

**Project administration:** Naoki Oishi.

**Supervision:** Marie N Shimanuki, Makoto Hosoya, Nobuyoshi Tsuzuki, Takanori Nishiyama, Hiroyuki Ozawa, Naoki Oishi.

**Writing – original draft:** Katsuyoshi Idei.

**Writing – review & editing:** Mitsuru Kitamura, Naoki Oishi.
